# A comparative study on shared-use medicines in Tibetan and Chinese medicine

**DOI:** 10.1186/s13002-019-0320-5

**Published:** 2019-08-23

**Authors:** Ming-ming Zhao, Ke-ru Wang, Rui Gu, Shi-hong Zhong

**Affiliations:** 1 Institute of Chinese Medicinal Science, State Key Laboratory of Quality Research in Chinese Medicine, University of Macau Avenida da Universidade, Taipa, Macau, China; 2 Chengdu University of Traditional Chinese Medicine, No. 1166, Liu-tai Road, Wenjiang District, Chengdu, 611173 China; 3 Chengdu Medical College, No. 783, Xing-du Road, Xingdu District, Chengdu, 610083 China

**Keywords:** Tibetan medicine, Traditional Chinese medicine, Shared-use medicines, Comparative study

## Abstract

**Background:**

Tibetan medicine (TM) and traditional Chinese medicine (TCM) are two independent traditional medical systems. Due to geographical factors, the development of Tibetan medicinal theory is relatively independent, but there are still many shared-use medicines in TM and TCM. However, a thorough and comparative study on those medicines is still absent. This study listed shared-use medicines by TM and TCM and analyzed the similarities and dissimilarities of these two medical systems. This paper also aimed to understand mutual influences like the shared history of TM and TCM and to roughly outline the exchanging process between them.

**Methods:**

Shared-use medicines in TM and TCM were listed alphabetically. Information on the scientific name, material name, medicinal parts, and medical efficacy were extracted from publications. Shared-use medicines were grouped according to medicinal properties and medicinal parts used by TM and TCM. The historical origin and current status of clinical prescriptions of shared-use medicines were analyzed.

**Results:**

A total of 136 shared-use medicines in TM and TCM were listed. Shared-use medicines that were used for a similar purpose in TM and TCM accounted for 14% of the total, while those used for different purposes accounted for 49% of the total, with some of the latter being commonly used in TCM. Shared-use medicinal herbs that originated from both Tibetan and Han regions accounted for 49% of the total, and those that were imported from South Asia and Southeast Asia were frequently observed in TM.

**Conclusion:**

Owing to its unique geographical location and cultural diversity, the Tibetan region played a role as a development cradle for various traditional medicinal theories and knowledge. Medicinal knowledge was exchanged between TM and TCM during their parallel independent growth. Shared-use medicines in TM and TCM were mostly determined by flora similarity and medicinal trade, and they marked significant differences in their medicinal properties. However, medicines that were used for similar purposes in TM and TCM presented obvious commercial medicinal characteristic as well as the same chemical profile. The Tibetan region not only provided medicinal usage knowledge of TCM, but also served as a supply of medicinal resources attributing to “high altitude” locations.

## Background

Tibetan medicine (TM) is rich in medicinal resources, approximately 80% of which are produced in the Qinghai-Tibet Plateau, with significant ethnic culture and regional biological distinctness, which is reflected in it being a culturally traditional medicine. Tibetan medicine presents obvious characteristics of national culture and distinct ecological-geographic conditions [1]. It is generally believed in medical documents that during the inheritance and development process, TM has continuously acquired knowledge and absorbed beneficial ingredients from Chinese traditional medicine (TCM), Indian Ayurveda medicine, and Arabic medicine. Earlier research on the history of Tibetan medicine [2] showed that Chinese and Tibetan medicines were widely exchanged during the Tubo period, while there are few documents about medical communication occurring between Han and Tibetan people at a later period. Princess Wencheng of the early Tang Dynasty brought “a hundred prescriptions for the treatment of four hundred and four diseases, five kinds of diagnostic methods, six kinds of medical devices, and four kinds of theory on medicine...” to Tibet, following her marriage with King Songtsan Gambo of Tibet. A Han doctor translated them into the *Medical Encyclopedia*, and later, traditional Chinese medicine (TCM) practitioner Han Wenhai contributed *Small Sporadic External Therapy* and *Fearless Weapons* coauthored with Scorpion and Persian Doctors during the period of Chi Dezuzan (704–745). Princess Jincheng dedicated a variety of medical books, such as *Jumbo Drugs* by Han doctor Zhang Song and *Human Body Torso* translated by doctor Zambia Laha from Yutian, in the Western Region, to the Tibetan king. During the period of Akamatsu Dezan, *Yuewang Drug Clinic* was translated into the Tibetan language, and three famous Chinese medical practitioners, including Dongsong Gangwa of the Tang Dynasty, were recruited. At the end of the eighth century, Yutuo Yuandan Gongbu was sent by the Tibetan King to the mainland, such as Wutai mountain, to study TCM [3].

Based on the above historical literature, many scholars believe that Tibetan medicine has been largely influenced by traditional Chinese medicine during its development. An earlier article [4] revealed that there were many connections in the historical background, theoretical system, and application of prescriptions between TM and TCM, and TM possesses its own unique features in addition to what it shares in common with TCM regarding medicinal properties, pharmacology, clinical medical practices, prescriptions, and patent medicines. However, we still do not know much about the similarity or dissimilarity of the two traditional medicinal systems, and a thorough study on shared-use medicines in TM and TCM is still absent. In view of this, the main goals of the present paper are to systematically list shared-use medicines in TM and TCM, evaluate the differences of the medicinal parts and medical efficacies, analyze the mutual influence of history and the exchanging process of the two medical systems, and discuss the historical and cultural background leading to those shared-use medicines.

## Methods

TCM use a total of 12,807 [5] different types of medicine, and TM use 3105 [6] species according to documentation. However, comprehensive statistics on these total varieties cannot reflect the clinical status of TM and TCM. Luckily, earlier, Zhong Guoyue compiled 502 medicinal materials that are used in the Tibetan medicine prescriptions. The present paper listed commonly used Tibetan medicines based on his study, including publications such as the *Ministry of Health of the People's Republic of China • Tibetan Medicine, Book 1* [7] and the *Tibetan Medicine Standards for Six Provinces* [8]. According to *The Collection of Chinese Herbal Medicines* [9], shared-use medicines, including animal, plant, mineral, and fungi resources, were documented alphabetically. Information on the scientific name, medicinal parts, and medical efficacy was extracted from publications, such as *Chinese Pharmacopoeia, Book 1 (2015 edition)* [10], *The Collection of Chinese Herbal Medicines* [9], *Chinese Medicine Dictionary* [11], *Ministry of Health of the People's Republic of China • Tibetan Medicine, Book 1* [7], *Tibetan Medicine Standards for Six Provinces* [8], *The Four-Part Medical Classics* [11], *Crystal Beads* [12], *Tibetan Medicine Chronicles* [13], *Chinese Materia Medica (Tibetan Medicine)* [14], *Tibetan medicine in China* [15], and *Tibetan Herbals in China* [16], and was pooled together. Information on the family and genus was analyzed based on *Flora Reipublicae Popularis Sinica* [17], and *Higher Plants of China* [18]. The shared-use medicines recorded by *Chinese Pharmacopoeia, Book 1 (2015 edition)* [10] and the *Medical Standards of the Ministry of Health of the People's Republic of China • Tibetan Medicine, Book 1* [7] were marked in this paper.

According to the medicinal property, the shared-use medicines in TM and TCM were divided into five categories: classes I, II, III, IV, and V. The shared-use medicines with the same purpose fell into class I; the shared-use medicines with medicinal usage by TM basically covering that by TCM fell into class II; the shared-use medicines with medicinal usage by TCM basically covering that by TM fell into class III; the shared-use medicines that were used for different purposes fell into class IV; and the shared-use medicines with partial overlapping medical effects in the two medicinal system fell into class V.

According to the medicinal part differences, the shared-use medicines were divided into five types: classes A, B, C, D, and E. The shared-use medicines with same medicinal parts in TM and TCM fell into class A; the shared-use medicines with medicinal parts in TM covering that in TCM fell into class B; the shared-use medicines with medicinal parts in TCM covering that in TM fell into class C; the shared-use medicines with totally different medicinal parts in the two medical systems fell into class D; and the shared-use medicines with partial overlapping medicinal parts in the two medicinal system fell into class E. The above information were given in Tables 1, 2, 3, 4 and 5.

**Table 1:** The Information of class I

No.	Family	Scientific name	Chinese name	Parts used in TCM	Parts used in TM	Uses in TCM	Uses in TM	Parts used	Disease groups treated in TCM	Disease groups treated in tm
1	Apidae	*Apis cerana* Fabr*	蜂蜜*	Honey	Beewax, honeybee	Indications: epigastric pain alleviated after meals or by pressing; dry cough; constipation; external use for sores, scalds, and burns [10]	Indications: dry cough without phlegm, intestinal dry constipation, counteract toxicity of aconitum; external use for ulcers in the mouth, sores, burns, and scalds [8]	B	Digestive, respiratory disorders	Digestive and respiratory disorders
2	Asteraceae	*Aucklandiae radix*	木香*	Root	Root	Indication: abdominal distension and epigastric pain, diarrhea, dysentery, indigestion, loss of appetite [10]	Indication: abdominal distension and epigastric pain, vomiting, diarrhea, and pneumonia [8]	A	Digestive disorders	Digestive disorders
3	Convolvulaceae	*Cuscuta chinensids* Lam.*	菟丝子*	Seed	Seed	Indications: impotence, seminal emission, dripping of urine after urination, enuresis, frequent urination, aching and weakness of the loins and knees, blurred vision and tinnitus; threatened abortion due to deficiency of the kidney; diarrhea due to hypofunction of the spleen and the kidney; an external use for vitiligo [10]	Indications: pain in the loins and knees, impotence, seminal emission, stranguria with turbid discharge, abnormal vaginal discharge, diarrhea, and tinnitus [14]	A	Reproductive system disorders	Reproductive system disorders
4	Elapidae	*Bungarus multicinctus* Blyth	银环蛇	Meat	Meat	Rheumatic, stroke, hemiplegia, convulsion, spasm, tetanus, leprosy, and scab [10]	Rheumatic, hemiplegia [19]; convulsion, spasm, tetanus, syphilis, and scab [13]	A	Nervous system ailments	Nervous system ailments
5	Fabaceae	*Dalbergia odorifera* T. Chen	降香*	Heartwood	Heartwood	Hematemesis, traumatic hemorrhinia, hypochondriac pain due to stagnation of liver qi, vomiting, and stomach pain [10]	Liver diseases, limb edema [13]	A	Circulatory system disorders	Circulatory system disorders
6	Piperaceae	*Piper longum* L*	荜拔*	Fruit	Fruit	Indications: epigastric pain, vomiting and diarrhea caused by cold, migraine; and external use for toothache [10]	Indications: rLung diseases in cold syndrome, precordial and abdominal pain with cold sensation, nausea and vomiting, borborygmus, and diarrhea [8]	A	Digestive disorders	Digestive disorders
7	Polygonaceae	*Rheum officinale* Baill.*	药用大黄*	Roots and rhizome	Roots and rhizome	Indications: fever with constipation, retention of feces and abdominal pain; dysentery; jaundice caused by damp-heat; hematemesis, epistaxis, inflammation of eyes and sore throat due to heat in blood; appendicitis with abdominal pain; boils, sores and abscess; amenorrhage due to blood stasis; traumatic injuries; hemorrhage from the upper gastrointestinal tract; and external use for scalds and burns [10]	Indications: constipation due to excessive heat, indigestion distension syndrome, tenesmus, jaundice in damp-heat syndrome, blood stasis, amenorrhea, sores, and boils [8]	A	Digestive disorders	Digestive disorders
8	Polygonaceae	*Rheum palmatum* L.*	掌叶大黄*	Roots and rhizome	Roots and rhizome	The same as above [10]	The same as above	A	Digestive disorders	Digestive disorders
9	Polygonaceae	*Rheum tanguticum* Maxim. ex Regel*	唐古特大黄*	Roots and rhizome	Roots and rhizome	The same as above [10]	The same as above	A	Digestive disorders	Digestive disorders
10	Rutaceae	*Zanthoxylum bungeanum* Maxim.*	花椒*《藏标》	Exocarp and seed	Exocarp and fruit	Indications: epigastric pain cold sensation, vomiting and diarrhea; abdominal pain due to intestinal parasitosis; ascariasis; and external use for itching in eczema [10]	Indications: gastropathy, fungi and trichomonad; external use for dermatosis [15]	E	Digestive, immune system ailments	Digestive and immune system ailments
11	Selaginellaceae	*Selaginella pulvinata* (Hook. et Grev.) Maxim.	垫状卷柏	Whole plant	Whole plant	Indications: amenorrhea, hematochezia, and archoptoma [10]	Indications: amenorrhea, masses in the abdomen, hemafecia, and prolapse of rectum [8]	A	Reproductive and digestive disorders	Reproductive and digestive disorders
12	ApiaceaeApiaceae	*Ferula sinkiangensis* K. M. Shen *	新疆阿魏*	Resin	Resin	Indications: indigestion, congestion, and stomach pain due to parasitic diseases [10]	Indications: indigestion, congestion, and stomach pain due to parasitic diseases [8]	A	Digestive disorders	Digestive disorders
13	ApiaceaeApiaceae	*Ferula fukanensis* K. M. Shen *	阜康阿魏*	Resin	Resin	Indications: indigestion, congestion, and stomach pain due to parasitic diseases [10]	Indications: indigestion, congestion, and stomach pain due to parasitic diseases [8]	A	Digestive disorders	Digestive disorders
14	Zingiberaceae	*Alpinia katsumadai* Hayata*	草豆蔻*	Seed pellets expelled	Seed pellets expelled	Indications: accumulation of damp-cold in the spleen and the stomach manifested by epigastric distention, and pain and cold feeling accompanied with belching, nausea, vomiting, and anorexia [10]	Indications: spleen diseases and gastropathy [15]	A	Digestive disorders	Digestive disorders
15	Zingiberaceae	*Alpinia officinarum* Hance*	高良姜*	Rhizome	Rhizome	Indications: epigastric pain with cold sensation; vomiting, belching, and acid regurgitation due to cold in the stomach [10]	Indications: epigastric pain with cold sensation, vomiting and diarrhea due to cold in the spleen and stomach; loss of appetite [8]	A	Digestive disorders	Digestive disorders
16	Zingiberaceae	*Kaempferia galanga* L.*	山柰*	Rhizome	Rhizome	Indications: dyspepsia accompanied with epigastric distension, pain, and cold feeling [10]	Indications: complication of badkan diseases and rlung diseases [15]	A	Digestive disorders	Digestive disorders
17	Zingiberaceae	*Amomum tsaoko* Crevost et Lemair	草果*	Fruit	Fruit	Indication: abdominal distension and epigastric pain, vomiting, malaria, and fever [10]	To remove cold in the spleen and stomach; promote digestion [13]	A	Digestive disorders	Digestive disorders
18	Zingiberaceae	*Zingiber officinale* Rosc.*	姜*	Rhizome	Rhizome	Indications: common cold caused by exterior wind-cold. Vomiting due to cold in the stomach [10]	Indications: badkan diseases, rlung diseases, abdomen pain due to cold in the spleen and stomach, vomiting and diarrhea, cough and dyspnea due to cold in the lung, and rheumatoid arthralgia [8]	A	Respiratory, digestive disorders	Respiratory, digestive, and immune system ailments
19	——	*Cordyceps*	冬虫夏草	Fungi	Fungi	Pulmonary tuberculosis, cough, hemoptysis, dyspnea of deficiency type, night sweating, emission, impotence, and soreness-tired of waist and knee [10]	Lung diseases, emission, and impotence [14]	A	Respiratory and reproductive system disorders	Respiratory and reproductive system disorders

*Shared-use medicines recorded by the 2015 edition of *China Pharmacopoeia*

Class A included the shared-use medicines with the same medicinal parts in TM and TCM; class B included the shared-use medicines with medicinal parts in TM covering that in TCM; class C included the shared-use medicines with medicinal parts in TCM covering that in TM; class D included the shared-use medicines with totally different medicinal parts in the two medical systems; and class E included the shared-use medicines with partial overlapping medicinal parts in the two medicinal systems

**Table 2:** Information on class II

No.	Family	Scientific name	Chinese name	Parts used in TCM	Parts used in TM	Uses in TCM	Uses in TM	Parts used	Disease groups treated in TCM	Disease groups treated in TM
1	Aristolochiaceae	Aristolochia debilis Sieb. et Zucc*	马兜铃*	Root	Aerial parts	Indications: dyspnea asthma, cough and bloody sputum due to heat in the lung; bleeding, and swollen and painful hemorrhoids due to heat in the large intestine [10]	Indications: blood disease, clung disease, liver disease, foorgan disease, badkan diseases, plague disease [15]	D	Respiratory and digestive disorders	Respiratory and digestive disorders
2	Cervidae	*Cervus elaphus* Linnaeus	马鹿	Antler	Antler and testis	Indications: impotence, emission, carbuncle, sores, and swelling [10]	Indications: vertigo, impotence, flaccid limbs, deafness, and metrorrhagia [15]	B	Reproductive and locomotor system ailments	Reproductive and urinary system disorders
3	Cervidae	*Cervus nippon* Temminck	梅花鹿	Antler	Antler and testis	Indications: impotence, emission, carbuncle, sores, and swelling [10]	Indications: vertigo, impotence, flaccid limbs, and deafness, metrorrhagia [15]	B	Reproductive and locomotor system ailments	Reproductive and urinary system disorders
4	Asteraceae	*Inula racemosa* J. D. Hooker *	土木香*	Roots	Roots	Indications: distending pain in the chest, hypochondria and epigastrium, vomiting and diarrhea; bruise or sudden sprain of the cheat with pain during breathing; abortion threat [10]	To remove heat from blood [12]	A	Digestive disorders	Immune system ailments and digestive disorders
5	Asteraceae	*Dolomiaea souliei* (Franchet) C. Shih	川木香	Roots	Roots	Indications: abdominal distension, gurgling sound, and diarrhea [10]	Indications: loss of appetite, gastric ulcer, abdominal distension, and rheumatism [15]	A	Digestive disorders	Digestive disorders
6	Crassulaceae	*Rhodiola crenulata*(Hook. f. et Thoms. )H. Ohba *	大花红景天*	Roots and rhizome	Roots and rhizome	Indications: constriction in the chest with heart pain, apoplexy, lassitude, and asthma [10]	Indications: nausea, vomiting, cyanosis on the lips and palm due to climatic sickness; loss of strength, chest distress, insomnia and dream-disturbed sleep, and also used for tuberculosis [14]	A	Digestive and nervous system ailments	Digestive, and nervous system ailments
7	Brassicaceae	*Raphanus sativus* L.	萝卜	Aerial parts	Roots	To promote digestion and stop thirst, remove heat, and counteract toxicity.	Indications: masses in the abdomen, obstinate phlegm, dyspepsia due to stomach cold, eye disease, consumptive thirst, constipation, and influenza [16]	D	Digestive disorders	Digestive disorders
8	Fabaceae	*Medicago ruthenica* (L.) Trautv.	花苜蓿	Seed	Whole plant	To remove toxic-heat, relieve cough, and arrest bleeding [9]	Indications: boils and measles, cough due to heat in the lung [7]. External use to eliminate inflammation and arrest bleeding [8]	B	Respiratory disorders	Respiratory disorders
9	Liliaceae	*Allium sativum* L.	大蒜	Bulb	Bulb	Indications: carbuncle, furuncles, skin disease, phthisis, cough, diarrhea, and dysentery [10]	Indications: carbuncle toxin, skin diseases, cold, hemorrhoids, urinary retention, and leprosy [14]	A	Respiratory disorders	Urinary , respiratory and digestive disorders
10	Malvaceae	*Malva crispa Linn.* *	冬葵*	Roots, stem, seed, and leaf	Flower and fruits	Indications: enuresis, edema, thirst, and urinary infection with oliguria [10]	Indications: anuresis, gonorrhea, edema, thirst, and seminal emission [7]	D	Urinary system disorders	Urinary, reproductive system disorders
11	Myristicaceae	*Myristica fragrans* Houtt.*	肉豆蔻*	Kernel	Kernel	Indications: deficiency-cold of the spleen and stomach with persistent diarrhea, epigastric and abdominal distension and pain, anorexia, and vomiting [10]	Indications: rheumatic heart disease, abdominal pain due to cold in the stomach, dyspepsia, feeling of anxiety in the qi deficiency syndrome, and epidemic febrile disease [16]	A	Digestive disorders	Circulatory system disorders
12	Myrtaceae	*Ewgewia caryophyllata* Thunb.*	丁香 *	Flower bud	Flower bud	Indications: hiccup, vomiting, diarrhea, and abdominal pain with cold sensation [10]	Indications: hiccup, vomiting, diarrhea, abdominal pain with cold sensation, and impotence [8]	A	Digestive disorders	Digestive disorders
13	Pedaliaceae	*Sesamum indicum* L.	胡麻《藏标》	Seed	Seed	To replenish the liver and kidney, tonify blood, moisten intestines, and promote lactation [10]	Indications: wind syndrome of head and dizziness due to yin deficiency of the liver and kidney, constipation in deficiency syndrome [8]	A	Reproductive and nervous system ailments and digestive disorders	Reproductive and nervous system ailments and digestive disorders
14	Sciuridae	*Trogopterus xanthipes*	复齿鼯鼠	Dry excrement	Excrement, meat	To activate blood circulation and eliminate blood stasis, and arrest bleeding [10]	Excrement: promote the flow of blood and dredge the meridians, eliminate blood stasis and pain, use for stomach pain, dysmenorrhea, and amenorrhea; meat: gynecological diseases, and oxytocin, contraception [16]	B	Digestive, reproductive and locomotor system ailments	Digestive, reproductive system disorders
15	Piperaceae	*Piper nigrum* L.*	胡椒*	Fruit	Fruit	Indications: vomiting, abdominal pain, diarrhea and loss of appetite due to cold in the stomach; inappetence, and epilepsy with profuse phlegm [10]	Indications: badkan diseases, cold phlegm, indigestion, vomiting and dysentery due to cold, and abdominal pain with cold sensation [8]	A	Digestive disorders	Digestive disorders
16	Ranunculaceae	*Aconitum pendulum* Busch	铁棒锤	Tuber	Tuber, seedling, roots	To dispel wind and relieve pain, remove blood stasis, arrest bleeding, reduce swelling, and remove toxin [9]	Indications: rlung diseases, cold diseases, yellow fluid diseases, leprosy, and epilepsy [7]	B	Locomotor system ailments	Nervous system ailments
17	Rubiaceae	*Rubia cordifolia* L.*	茜草*	Roots	Roots, rhizome, and whole plant	Indications: spitting of blood, epistaxis, abnormal uterine bleeding, traumatic bleeding; amenorrhea, arthralgia, and traumatic swelling and pain [10]	Indications: whole grass: pneumonia, nephritis and trichomonal vaginitis; root; spitting of blood, epistaxis, hematochezia, abnormal uterine bleeding, menstrual disorders, menoxenia abdominal pain, ecchymoma pain, trauma injury, and bloody dysentery [12]	B	Digestive, locomotor system ailments	Digestive, reproductive and locomotor system ailments
18	Euphorbiaceae	*Euphorbia fischeriana* Steudel *	狼毒*	Roots	Roots	To cause urination, remove indigestion, and kill parasites [10]	Indications: boils and sores, scrofula, external use for dermatitis, and ulceration [7]	A	Immune system ailments	Respiratory and immune system ailments
19	ApiaceaeApiaceae	*Coriandrum sativum* L.	芫荽	Fruits	Fruits	Indications: measles, cold, indigestion, and loss of appetite [9]	Indications: indigestion, loss of appetite, thirst, gastric ulcer, measles, cold, stomach diseases, and dysentery [7]	A	Digestive disorders	Digestive disorders
20	Zingiberaceae	*Amomum kravanh* Pierre ex Gagnep	白豆蔻	Seed	Fruits	To promote the flow of qi and arrest vomiting, warm the stomach, and promote digestion [10]	Indications: heart disorder, gastropathy, and nephropathy characterized by cold [16]	A	Digestive disorders	Digestive, urinary system disorders
21	Zingiberaceae	*Alpinia galanga* (L.) Willd	大高良姜	Fruits	Fruits, rhizome	To stimulate the functional activity of the stomach by expelling cold, promote the flow of qi, and relieve pain [10]	Indications: fruit; nephropathy, gastropathy [15] Rhizome; precordial and abdominal pain with cold sensation; indigestion due to stomach-cold, loin pain in kidney deficiency syndrome, and lung abscess [16]	B	Digestive disorders	Digestive, respiratory disorders

*Shared-use medicines recorded by the 2015 edition of *China Pharmacopoeia*

Class A included the shared-use medicines with the same medicinal parts in TM and TCM; class B included the shared-use medicines with medicinal parts in TM covering that in TCM; class C included the shared-use medicines with medicinal parts in TCM covering that in TM; class D included the shared-use medicines with totally different medicinal parts in the two medical systems; and class E included the shared-use medicines with partial overlapping medicinal parts in the two medicinal systems

**Table 3:** Information on class III

No.	Family	Scientific name	Chinese name	Parts used in TCM	Parts used in TM	Uses in TCM	Uses in TM	Parts used	Disease groups treated in TCM	Disease groups treated in TM
1	Thymelaeaceae	*Stellera chamaejasme* Linn.*	瑞香狼毒*	Roots	Roots	To cause urination, remove indigestion, and kill parasites [10]	Indications: boils and sores, scrofula, external use for dermatitis, and ulceration [7]	A	Digestive disorders	Immune system ailments
2	Juglandaceae	*Juglans regia* L.	胡桃	Kernel, exocarpand leaf	Exocarp and leaf	Kernel: to reinforce kidney, relieve asthma, use for tinnitus, cough, and asthma in kidney deficiency syndrome, seminal emission, lumbago, tympanitis, and astriction; Exocarp: relieve swelling and itching, tracheitis, lepra alphos, tinea capitis, sores, and boils; Leaf: leucorrhea [10]	Indications: rlung diseases, spasm of tendons and collaterals, aching and weakness of the loins and knees, constipation, seminal emission, and impotence [8]	C	Respiratory and reproductive system disorders	Reproductive and digestive disorders
3	Fabaceae	*Glycyrrhiza inflata* Bat.	胀果甘草*	Rhizome and root	Rhizome and root	Indications: hypofunctioning of spleen and stomach, cough, palpitation, swollen sore throat, and sores [10]	Indications: lung diseases [15]	A	Digestive and respiratory disorders	Respiratory disorders
4	Fabaceae	*Glycyrrhiza uralensis* Fisch.	甘草*	Rhizome and root	Rhizome and root	Indications: hypofunctioning of spleen and stomach, cough, palpitation, swollen sore throat, and sores [10]	Indications: lung diseases [15]	A	Digestive and respiratory disorders	Respiratory disorders
5	Leguminosae	*Trigonella foenum-grecum* L.	葫芦巴	Seed	Seed	Indications: cold syndrome of the kidney due to yang deficiency marked by pain and coldness in the lower abdomen; hernia; and weakness and edema of the legs caused by cold-damp [10]	Indications: mass formation in the abdomen, discomfort and distension in the chest and hypochondriac regions, and *kakke* due to cold-dampness [8]	A	Reproductive system disorders and digestive disorders	Digestive disorders
6	Liliaceae	Polygonatum sibiricum Redouté*	黄精*	Rhizome	Rhizome	Indications: weakness of the spleen and the stomach marked by lassitude, dryness in the mouth and anorexia; dry cough due to deficiency of yin of lung; deficiency of vital essence and blood; and wasting-thirst caused by internal heat [10]	Indications: various deficiency, dry cough hydrodipsia, and thirst [8]	A	Digestive and respiratory disorders	Respiratory disorders
7	Arecaceae	Areca catechu L*	槟榔*	Exocarp	Seed	Indications: taeniasis, ascariasis, and fasciolopsiasis; abdominal pain due to intestinal parasitosis; diarrhea and tenesmus due to accumulation of undigested food; edema and weakness of the legs; and malaria [10]	Indications: kidney disease, toothache, and parasitic diseases [14]	D	Digestive and urinary system disorders	Reproductive and digestive disorders
8	Polygonaceae	*Rumex nepalensis* Spreng.	尼泊尔酸模	Roots and leaf	Roots	To remove toxic-heat, arrest bleeding, relax bowels, and kill fungi and trichomonad [10]	Indications: boils and eczema [7]	C	Digestive disorders and immune system ailments	Immune system ailments
9	Ranidae	*Rana chensinensis*	中国林蛙	Meat	Meat	Indications: palpitation, insomnia, night sweating, and hemoptysis [10]	Indications: neurasthenia [12]	B	Respiratory disorders	Urinary and nervous system ailments
10	Ranunculaceae	*Coptis chinensis* Franch.	黄连*	Rhizome	Rhizome	Indications: vomiting, diarrhea, jaundice, hyperthermia, insomnia due to restlessness, toothache, thirst, skin diseases, and eczema [10]	Indications: infectious disease, anthrax, dysentery, and incised wound [13]	A	Digestive disorders	Immune system ailments
11	Rutaceae	*Zanthoxylum simulans* Hance.	野花椒	Root and fruit	Exocarp	To dispel cold from the spleen and stomach, relieve pain, kill fungi and trichomonad, and prevent impregnation [10]	Indications: gastropathy, fungi and trichomonad, and external use for dermatosis [15]	D	Digestive and immune system ailments	Digestive and immune system ailments
12	Solanaceae	*Lycium chinense* Mill.*	枸杞*	Root and bark	Fruits	Indications: general debility with deficiency of vital essence with manifestations of aching of the loin and knees, dizziness, and tinnitus; diabetes by internal heat; anemia; and impaired vision [10]	Indications: heart febrile disease and gynecopathy [15]	D	Reproductive system disorders	Circulatory system disorders and respiratory ailments
13	Urticaceae	*Urtica laetevirens* Maxim.	宽叶荨麻	Whole plant, roots, and seed	Aerial parts	Indications: rheumatism arthralgia, postpartum and infantile convulsion, infantile paralytic sequel, hypertension, dyspepsia, stool atresia. External use for urticaria initially and snake bite [10]	Indications: chronic heart diseases and dyspepsia due to rlung diseases [7]	C	Locomotor, nervous system ailments and digestive disorders	Digestive disorders
14	——	Sulfur*	硫黄*	Natural element sulfur minerals	Natural element sulfur minerals	Indications: external use for scabies and favus, abscesses due to cold and phlegm retention and malignant ulcers, oral administration for impotence with cold lower extremities, and asthma or constipation of deficiency-cold type [10]	Indications: carbuncles, sores and boils, tetter, and leprosy and external use for mange, malignant sore, pruritus [15]	A	Reproductive and digestive disorders	Circulatory system disorders and immune system ailmentailments

*Shared-use medicines recorded by the 2015 edition of *China Pharmacopoeia*

Class A included the shared-use medicines with the same medicinal parts in TM and TCM; class B included the shared-use medicines with medicinal parts in TM covering that in TCM; class C included the shared-use medicines with medicinal parts in TCM covering that in TM; class D included the shared-use medicines with totally different medicinal parts in the two medical systems; and class E included the shared-use medicines with partial overlapping medicinal parts in the two medicinal systems

**Table 4:** Information on class IV

No.	Family	Scientific name	Chinese name	Parts used in TCM	Parts used in TM	Uses in TCM	Uses in TM	Parts used	Disease groups treated in TCM	Disease groups treated in TM
1	Acanthaceae	*Adhatoda vasica* Nees	鸭嘴花	Whole plant	Twig	To dispel wind and activate blood circulation; eliminate blood stasis and relieve pain; and re-joint the bone [10]	Indications: blood and liver heat-related diseases, *mkhris pa* diseases, traumatic injury, boils, swelling, and pain [15]	A	Locomotor system ailments	Digestive, locomotor system ailments
2	Araceae	*Acorus calamus* L	菖蒲	Rhizome	Rhizome	To dispel wind to resolve the exterior, clear heat and remove dampness, relieve cough resolve phlegm, eliminate blood stasis, and reduce swelling [10]	Indications: dyspepsia, diphtheria, boils, and sores [7]	A	Respiratory and locomotor system ailments	Digestive disorders
3	Berberidaceae	*Sinopodophyllum hexandrum* (Royle) Ying.	桃儿七	Roots and rhizome	Fruits	Indications: rheumatic arthritis, traumatic injury, cough due to wind-cold, and menoxenia [10]	Indications: stasis syndrome of women, fetal death, mazischesis, and amenorrhea [8]	D	Immune system ailments, locomotor, respiratory, and reproductive system disorders	Reproductive system disorders
4	Bombacaceae	*Gossampinus malabarica* (DC.)Mern	木棉花*	Flower	Flower	Indications: diarrhea, dysentery, and hemorrhoids [10]	Indications: lung and liver diseases [15]	A	Digestive disorders	Digestive disorders
5	Bovidae	Bos taurus domesticus Gmelin *	牛黄*	Dry gallstones	Dry gallstones	Indications: impairment of consciousness in febrile diseases and stroke; infantile convulsion, epilepsy, mania; sore throat, ulcers in the mouth; carbuncles, and boils [10]	Indications: plague epidemic disease and liver-heat syndrome [11]	A	Nervous system ailments and respiratory disorders	Digestive disorders
6	Burseraceae	*Boszvellia carterii* Birdw *	乳香树*	Resin	Resin	Indications: stomach pain, dysmenorrhea, amenorrhea, rheumatism, traumatic injury, carbuncle, and sore [10]	Indications: skin diseases [14]	A	Circulatory system disorders and locomotor system ailments	Immune system ailments
7	Caryophyllaceae	*Arenaria kansuensis* Maxim	甘肃蚤缀	Whole plant	Whole plant	To nourish yin and tonify blood, replenish the kidney, and reinforce the bone [10]	Indications: pneumonia and various lung diseases [7]	A	Digestive disorders	Respiratory disorders
8	Cervidae	*Moschus berezovskii* Flerov	林麝	Secretions in sweet bursa of male body	Secretions in sweet bursa of male body, meat, testis, feces	To restore consciousness and activate blood circulation, stimulate menstruation, reduce swelling, and relieve pain [10]	Indications: stroke, syncope due to phlegm, sudden attack of precordial and abdominal pain, kidney disease, masses in the abdomen; external use for traumatic injury, carbuncle-abscess, and furuncles [15]	B	Nervous, circulatory and locomotor system ailments	Nervous system ailments, locomotor, reproductive, and digestive disorders
9	Cervidae	*Moschus moschiferus* Linnaeus	原麝	Secretions in sweet bursa of male body	Secretions in sweet bursa of male body, meat, testis, and feces	To restore consciousness and activate blood circulation, stimulate menstruation, reduce swelling, and relieve pain [10]	Indications: stroke, syncope due to phlegm, sudden attack of precordial and abdominal pain, kidney disease, masses in the abdomen; external use for traumatic injury, carbuncle-abscess, and furuncles [15]	B	Nervous, circulatory and locomotor system ailments	Nervous, locomotor, reproductive, and digestive disorders
10	Cervidae	*Mochus sifanicus* Buchner	马麝	Secretions in sweet bursa of male body	Secretions in sweet bursa of male body, meat, testis, and feces	To restore consciousness and activate blood circulation, stimulate menstruation, reduce swelling, and relieve pain [10]	Indications: stroke, syncope due to phlegm, sudden attack of precordial and abdominal pain, kidney disease, masses in the abdomen; external use for traumatic injury, carbuncle-abscess, and furuncles [15]	B	Nervous, circulatory and locomotor system ailments	Nervous system ailments, locomotor, reproductive, and digestive disorders
11	Combretaceae	*Terminalia chebula* Retz. *	诃子*	Fruit	Fruit	Indications: protracted diarrhea with hematochezia or prolapse of the rectum and chronic cough with sore throat and hoarseness [10]	Indications: rlung diseases, blood diseases, *mkhris pa* diseases, and badkan diseases [15]	A	Digestive, urinary, and respiratory disorders	Digestive and circulatory system disorders
12	Combretaceae	Terminalia chebula Retz. var. tomentella (Kurz) C. B. Clarke	绒毛诃子	Fruit	Fruit	To check diarrhea and chronic cough and subdue the upward qi [10]	Indications: rlung diseases, blood diseases, *mkhris pa* diseases, and badkan diseases [15]	A	Digestive and reproductive system disorders	Digestive disorders
13	Asteraceae	*Carthamus tinctorius* L. *	红花*	Flower	Flower	Indications: amenorrhea, dysmenorrhea; retention of lochia; abdominal masses; traumatic injuries, sores, and ulcers with swelling and pain [10]	Indications: pneumonia, hepatitis, blood heat, carbuncles, traumatic injury, and gynecopathia [14]	A	Reproductive and locomotor system ailments	Respiratory, locomotorand reproductive system disorders
14	Asteraceae	*Saussurea laniceps* Hand.-Mazz.	绵头雪莲花	Whole plant	Whole plant	To tonify kidney and reinforce yang; regulate menstruation by arresting bleeding [9]	Indications: head trauma, anthrax, pricking pain, gynecopathy, rheumatic arthritis, and stroke. External use for swelling [7]	A	Reproductive system disorders	Reproductive and immune system ailments
15	Cucurbitaceae	*Lagenaria siceraria* (Molina) Standl.	葫芦	Exocarp, seed	Seed	To cause urination, reduce swelling. Used in edema, ascites, and tuberculous cervical lymphadenitis [9]	Indications: dysentery due to heat, pulmonary disease, and rash [7]	C	Urinary and respiratory disorders	Respiratory and immune system ailments
16	Dipterocarpaceae	*Dipterocarpus turbinatus* Gaertn.f. *	龙脑香*	Resin	Resin	Indications: loss of consciousness in stroke and attack of noxious factors, syncope due to violent excitement or postpartum anemia; inflammation of eyes, aphtha, and swollen sore throat [10]	Indications: high fever and chronic hotness fever [14]	A	Nervous system ailments and respiratory disorders	Respiratory disorders
17	Equidae	*Equus asinus* L.	驴	Skin	Blood, meat	Indications: hemostasis [10]	Indications: rheumatism [14]	D	Circulatory system disorders	Nervous system ailments
18	Gentianaceae	*Gentiana crassicaulis* Duthie ex Burk *	粗茎秦艽*	Roots	Flower, roots	Indications: rheumatic or rheumatoid arthritis with muscular contracture and severe joint pain; fever recurring in the afternoon, and fever in infants with malnutrition [10]	Indications: tonsillitis, urticaria, carbuncle, and rheumatoid arthritis [13]	B	Nervous, locomotor system ailments	Respiratory and immune system ailments
19	Gentianaceae	*Gentiana straminea* Maxim	麻花艽	Roots	Flower, whole plant	To dispel wind and dampness and subdue deficient heat [10]	Indications: gastroenteritis, hepatitis, and cholecystitis [7, 8]	B	Respiratory ailments	Digestive disorders
20	Gramineae	Bambusae Concretio Silicea*	天竺黄	Secretion	Secretion	Indications: coma, stroke, and epilepsy and convulsion in children [10]	Indications: lung diseases [8]	A	Nervous system ailments	Respiratory disorders
21	Gramineae	Saccharum sinense Roxb.	甘蔗加工成红糖	Stem	Stem	Indications: blood stasis symptom [10]	Indications: diarrhea, impotence [14]	A	Digestive disorders	Reproductive system disorders
22	Iridaceae	*Crocus sativus* L.	藏红花*	Stigma	Stigma	Indications: amenorrhea, abdominal mass, and palpitation due to fright [10]	Indications: pneumonia and liver diseases [13]	A	Circulatory system disorders	Digestive disorders
23	Lamiaceae	*Lagopsis supine*(Steph. ex Willd.)Ikonn.-Gal. ex Knorr	夏至草	Whole plant	Flower, aerial parts and seed	To regulate menstruation by nourishing blood [9]	Indications: blood diseases due to heat, bloodshot eyes of nebula due to blood heat, parasitosis [15]	C	Nervous, reproductive system disorders	Immune system ailments
24	Lamiaceae	*Leonurus japonicus* Thunb.( Leonurus japonicas Houtt.)*	益母草*	Whole plant, fruits	Fruits, seed and aerial parts	Indications: menstrual disorders, dysmenorrhea, amenorrhea, incessant dripping of lochia; edema and oliguria such as edema in acute nephritis [10]	Indications: Fruits: enoxenia, amenorrhea, dysmenorrhea, masses formation in the stomach, conjunctival congestion edema pain, eye inflammation, corneal opacity, and hypertension [8]; aerial parts; Seed: blood diseases due to heat, bloodshot eyes of nebula due to blood heat, and parasitosis [15]	C	Reproductive and urinary system disorders	Reproductive and circulatory system disorders
25	Lamiaceae	*Leonurus sibiricus* L.	细叶益母草	Whole plant	Aerial parts and seed	Indications: menoxenia, amenorrhea, postpartum congestion abdominal pain, nephritis edema, dysuria, and hematuria [10]	Indications: blood diseases due to heat, bloodshot eyes of nebula due to blood heat, and parasitosis [15]	C	Reproductive and urinary system disorders	Immune system ailments
26	Fabaceae	*Acacia catechu* (L.f.)Willd*	儿茶*	Twig	Wood	Indications: festering wound difficult to heal up, skin diseases with watery discharge, ulcers in the mouth, and traumatic injury with pain and bleeding [10]	Indications: cough and thirst, external use for skin diseases with watery discharge, ulcerative gingivitis, and ulcers in the mouth and hemorrhoid [8]	A	Immune, locomotor system ailments	Respiratory and immune system ailments
27	Leguminosae	*Cassia tora* Linn*.*	决明*	Seed	Seed	Indications: headache and vertigo, eye diseases, and constipation [10]	Indications: skin diseases and epilepsy [15]	A	Five sense organs related ailments	Immune system ailments
28	Fabaceae	*Pterocarpus indicus* Willd.	紫檀	Heartwood	Heartwood	Indications: furuncle and swollen [20]	Indications: hypertension, pneumonia, and heart diseases [7]	A	Immune system ailments	Immune system ailments and respiratory disorders
29	Fabaceae	*Abrus precatorius* L	相思子	Roots, rattan cane, leaf, and seed	Seed	Roots, rattan: sore throat, and hepatitis. Leaf: bronchitis, seed external use for boils, and eczema [9]	Indications: gynecopathy and gallbladder masses [15]	C	Respiratory an immune system ailments	Reproductive system disorders
30	Liliaceae	*Fritillaria cirrhosa* D.Don *	川贝母*	Bulb	Bulb	Indications: dry cough due to heat in the lung and cough with bloody sputum in consumptive diseases [10]	Indications: yellow fluid diseases, menometrorrhagia, and trachitis [13]	A	Respiratory disorders	Locomotor and respiratory disorders
31	Loganiaceae	*Strychnos nux-vomica* Linn*.* *	马钱*	Seed	Seed	Indications: protracted arthritis, rheumatoid arthralgia; numbness and paralysis; sequela of poliomyelitis; traumatic injury; boils and sores [10]	Indications: rlung diseases, blood heat diseases, stomach cramps toxicosis [15]	A	Nervous system ailments, locomotor, immune system ailments	Circulatory system disorders
32	Malvaceae	*Abelmoschus moschatus* Medic	黄葵	Roots, leaf, and flower	Leaf, flower, and seed	Indications: Root: high fever, cough due to lung heat, postpartum milk atresia, stool constipate, dysentery, and urinary calculi. Leaf: topical fester swelling, felon, bone fracture. Flower: burns and scalds [9]	Indications: yellow fluid diseases, dermatosis, parasitosis, and itching [16]	E	Respiratory and digestive, urinary system disorders	Immune system ailments
33	Menispermaceae	*Tinospora sinensis* (Lour.) Merr.	中华青牛胆	Rattan	Stem	To soothe tendons and activate collaterals; dispel wind and relieve pain [10]	Indications: lung disease, rheumatoid arthritis [7]	D	Respiratory, digestive disorders	Digestive, immune system ailments
34	Oleaceae	*Fraxinus stylosa* Lingelsh*.*	宿柱白蜡树	Bark	Bark	Indications: diarrhea, leukorrhea, and conjunctive congestion with swelling and pain [10]	Indications: fracture, hyperosteogeny, and osteomyelitis [14]	A	Urinary system disorders	Locomotor system ailments
35	Orchidaceae	*Dendrobium hookerianum* Lindl.	金耳石斛	Stem	Aerial part	Indications: thirst, hiccup, and lassitude in the loin and legs [10]	Indications: indigestion, gastric ulcer, sore throat, and hemorrhoids [12]	B	Digestive disorders	Digestive disorders
36	Phytolaccaceae	*Phytolacca acinosa* Roxb.*	商陆*	Root	Root	Indications: anasarca with oliguria and constipation; external use for carbuncles and sores [10]	Indications: febrile disease, edema, distension, dysuria; external use for carbuncles, boils, swelling, and toxicity [15]	A	Digestive, urinary system disorders	Digestive, urinary system disorders
37	Polypodiaceae	*Pyrrosia lingua* (Thunb.) Farw.*	石韦*	Whole plant	Whole plant	Indications: urinary infection and urination; spitting of blood, epistaxis, hematuria, and abnormal uterine bleeding; and cough and asthma due to heat in the lung [10]	Indications: pus and sores in the chest, cough due to heat in the lung. Laryngopharyngitis, spinal cord cavity disease, traumatic injury, trauma hemorrhage, seminal emission in kidney deficiency syndrome, nephritis, edema, and urinary tract infections [16]	A	Urinary and respiratory disorders	Respiratory, locomotor, and reproductive system disorders
38	Pteriidae	Pinctada martensii( Dunker) *	马氏珍珠贝*	Pearl and dry shell	Pearl and dry shell	Indications: Pearl: palpitation and insomnia; convulsion, epilepsy; nebula; skin ulcerations difficult to heal. Nacre: headache, dizziness, fidgetingness, and insomnia; inflammation of the eyes due to heat in the liver; and blurred vision in deficiency of the liver [10]	Indications: commotio cerebri, head injury, white veins disease, numbness and pain in arthritis, and nosotoxicosis [14]	A	Nervous system ailments	Nervous system ailments
39	Lythraceae	*Punica granatum* L.*	石榴	Root, stem, bark, flower, leaf, fruit	Seed	Indications: protracted diarrhea, chronic dysentery; hematochezia, prolapse of the rectum; abnormal uterine bleeding, leukorrhagia; and intestinal parasitosis with abdominal pain [10]	Indications: anorexia, dyspepsia, and aching of kidney and loins due to cold in the stomach [14]	D	Digestive and reproductive system disorders	Digestive disorders
40	Ranunculaceae	*Paeonia veitchii* Lynch*	川赤芍*	Roots	Roots	Indications: maculation in epidemic diseases; spitting of blood, epistaxis; inflammation of the eye; pain in the chest; amenorrhea, dysmenorrhea; mass formation in the abdomen; traumatic injuries; and boils and sores [10]	Indications: carbuncles, fever. Flower: dermatosis, dermatitis [16]	B	Immune system ailments and reproductive and locomotor system ailments	Immune system ailments
41	Santalaceae	*Santalum album* L.*	檀香*	Heartwood	Heartwood	Indications: pectoral and abdominal pain due to stagnation of qi with cold; epigastric pain, loss of appetite; and angina pectoris in heart disease [10]	Indications: pneumonia and lung abscess [16]	A	Digestive and circulatory system disorders	Circulatory and respiratory system disorders
42	Sapindaceae	*Sapindus mukorossi* Gaertn.	无患子	Root, fruit	Seed	To remove heat and phlegm, check diarrhea and blood stasis [9]	Indications: diphtheria, vesicula seminalis disease, stranguria with turbid discharge, and frequent urination [7]	D	Respiratory disorders	Respiratory and urinary system disorders
43	Saxifragaceae	*Bergenia purpurascens* (Hook. f. et Thoms.) Engl *	岩白菜*	Rhizome	Rhizome, whole plant	To remove toxic heat, arrest bleeding, and regulate menstruation [9]	Epidemic febrile diseases, liver and lung heat diseases, and dysentery [15]	A	Circulatory system disorders	Respiratory and digestive disorders
44	Styracaceae	*Styrax benzoin* Dryand. *	安息香*	Resin	Resin	Indications: loss of consciousness in stroke and attack of noxious factors, syncope due to violent excitement or postpartum anemia; pain in the chest and epigastrium; infantile convulsion [10]	Indications: rlung diseases, subcutaneous ulcer, and boils [15]	A	Nervous system ailments and circulatory system disorders	Digestive, immune system ailments
45	ApiaceaeApiaceae	*Angelica sinensis* (Oliv.) Diels.*	当归*	Roots	Roots	Indications: anemia with dizziness and palpitation; menstrual disorders, amenorrhea, dysmenorrhea; constipation; rheumatic arthralgia; traumatic injuries; carbuncles, boils, and sores. Radix Angelicae Sinensis (stri-baked with wine) amenorrhea, dysmenorrhea, rheumatic, arthralgia, and traumatic injuries [10]	Indications: chronic febrile diseases, cardiopyretic disease, toxicosis, and complication of badkan diseases and rlung diseases [15]	A	Reproductive, digestive, immune, and locomotor system ailments	Digestive and respiratory disorders
46	ApiaceaeApiaceae	*Heracleum candicans* Wall. ex DC..	白亮独活	Roots	Roots	Indications: rheumatism and pain in the waist and knee [10]	Indications: boils, sores, and leprosy [15]	A	Immune system ailments	Immune system ailments
47	ApiaceaeApiaceae	*Notopterygium forbesii* de Boiss.	宽叶羌活	Roots and rhizome	Roots and rhizome	Indications: common cold caused by exterior wind-cold, rheumatism numbness, urticaria, and itching [10]	Indications: plague, greenfly and pinworm, hemorrhage disease, constipation, and leprosy [14]	A	Respiratory and immune system ailments	Immune system ailments and circulatory system disorders
48	Apiaceae	*Notopterygium incisum* Ting ex H. T. Chang*	羌活*	Roots and rhizome	Roots and rhizome	Indications: headache in common cold, rheumatic arthralgia, and aching of the back and shoulders [10]	Indications: leprosy, headache, laryngopathy, rheumatic arthralgia, and epidemic disease or cholera [15]	A	Respiratory and immune system ailments	Immune system ailments and circulatory system disorders
49	Unionidae	*Cristaria plicata* (Leach)*	褶纹冠蚌*	Pearl and dry shell	Pearl and dry shell	Indications: pearl: palpitation and insomnia; convulsion, epilepsy; nebula; skin ulcerations difficult to heal. Nacre: headache, dizziness, fidgetingness and insomnia; inflammation of the eyes due to heat in the liver; and blurred vision in deficiency of the liver [10]	Indications: commotio cerebri, head injury, white veins disease, numbness and pain in arthritis, nosotoxicosis [14]	A	Nervous system ailments	Nervous system ailments
50	Unionidae	*Hyriopsis cumingii*(Lea).*	三角帆蚌*	Pearl and dry shell	Pearl and dry shell	Indications: pearl: palpitation and insomnia; convulsion, epilepsy; nebula; skin ulcerations difficult to heal. Nacre: headache, dizziness, fidgetingness and insomnia; inflammation of the eyes due to heat in the liver; and blurred vision in deficiency of the liver [10]	Indications: commotio cerebri, head injury, white veins disease, numbness and pain in arthritis, and nosotoxicosis [14]	A	Nervous system ailments	Nervous system ailments
51	Valerianaceae	*Nardostachys chinensis* Batal.*	甘松*	Roots and rhizome	Roots and rhizome	Indications: epigastric and abdominal distension with anorexia and vomiting; and external use for toothache and swollen feet [10]	Indications: accumulation of damp-cold in the spleen and the stomach manifested by epigastric distention and pain, external use for ulcerative gingivitis, and dental caries and swollen feet [8]	A	Digestive disorders	Digestive disorders
52	Valerianaceae	*Nardostachys jatamansi* (D. Don) DC.	匙叶甘松	Roots and rhizome	Roots and rhizome	To regulate the flow of qi and relieve pain and to invigorate the spleen function [10]	To remove toxic heat, dispel cold and eliminate swelling [14]	A	Digestive disorders	Immune system ailments
53	Vitaceae	*Vitis vinifera* L.	葡萄	Fruits and roots	Fruits	Indications: measles, dysuria, rheumatism, and fracture [9]	Indications: lung diseases [15]	C	Respiratory, urinary , and locomotor system ailments	Respiratory disorders
54	Zingiberaceae	*Curcuma longa* L.*	姜黄*	Rhizome	Rhizome	Indications: pricking pain in the chest and hypochondriac regions, menorrhea, mass formation in the abdomen, rheumatic pain of the shoulders and arms, and traumatic swelling and pain [10]	Indications: ulceration and hemorrhoids, sores, and epidemic diseases [14]	A	Reproductive, immune, and locomotor system ailments	Immune system ailments
55	Zygophyllaceae	*Tribulus terrestris* L.*	蒺藜*	Fruits, whole plant	Fruits	Indications: headache and dizziness, distending pain in the hypochondrium; cessation of lactation, mastitis, and bloodshot eyes of nebula; and urticaria with itching [10]	Indications: headache, itching, distending pain in the hypochondrium; reverse of qi, inflammation of eyes, corneal opacity, masses formation in the abdomen, and cessation of lactation [8]	A	Digestive, reproductive and immune system ailments	Urinary system disorders
56	–	Calamina	炉甘石	Natural mineral	Natural mineral	Indications: eye diseases and pruritus [10]	Indications: skin diseases [12]	A	Immune system ailments	Digestive disorders
57	–	Realgar	雄黄*	Natural mineral	Natural mineral	Indications: carbuncle, furunculosis, snake bite, epilepsy, and malaria [10]	Indications: gall and diphtheria [13]	A	Immune system ailments and digestive disorders	Immune system ailments
58	–	Actinolite asbestos.	(阳起石)石棉	Natural mineral	Natural mineral	Indications: pain in waist and knee and impotence [20]	Indications: tendon injury, cough, swollen sore throat, and dysuria [14]	A	Reproductive system disorders	Locomotor and respiratory disorders
59	–	Calcitum*	寒水石*	Sulfate minerals	Sulfate minerals	Indications: fever and polydipsia, swollen sore throat, ulcers in the mouth on the tongue, toothache, and external use for burns and scalds [9]	Indications: various gastropathy and gastric ulcer due to dyspepsia, masses in the abdomen, edema, diarrhea, and trauma [14]	A	Respiratory disorders	Digestive disorders
60	–	Cinnabaris*	朱砂*	The sulfide minerals of cinnabar	The sulfide minerals of cinnabar	Indications: palpitation, insomnia and dream-disturbed sleep; epilepsy, mania, and infantile convulsion; blurred vision; ulcers in the mouth; painful swelling of the throat; and boils and sores [10]	Indications: pus of wounds, yellow fluid diseases, inflammation, and bone fracture [21]	A	Nervous system ailments and respiratory disorders	Locomotor system ailments
61	–	Gypsum Fibrosum	石膏	Natural mineral	Natural mineral	Indications: heal sore and promote granulation [10]	Indications: thirst, coma, delirium, heat stroke, dyspnea, headache, and toothache [22]	A	Immune system ailments	Respiratory disorders
62	–	Hematitum	刚玉族赤铁矿	Natural mineral	Natural mineral	Indications: vertigo and tinnitus, vomiting, hiccup, hematemesis, and metrorrhagia [10]	Indications: fracture, traumatic injury [14]	A	Digestive disorders	Locomotor system ailments
63	–	Magnetitum *	磁石*	The oxide minerals spinel clan	The oxide minerals spinel clan	Indications: dizziness, blurring of vision, tinnitus, impairment of hearing, palpitation, insomnia, and dyspnea due to diminished function of the kidney [10]	Indications: traumatic injury of head [12]	A	Nervous system ailments	Locomotor system ailments
64	–	Natrii sulfas	芒硝	Natural mineral	Natural mineral	Indications: constipation, pruritus, alopecia [10]	Indications: indigestion, constipation, edema, heart diseases, tumor, and jaundice [14]	A	Digestive disorders	Digestive disorders
65	–	Pyritum*	自然铜*	Pyrite sulphide minerals	Pyrite sulphide minerals	Indications: traumatic swelling and pain, and bone fracture [10]	To benefit brain and the liver [12]	A	Locomotor system ailments	Nervous system ailments
66	–	Succinite (Amber)	琥珀	Resin	Resin	Indications: infantile convulsion, epilepsy, diseases, palpitation, insomnia, dysuria, urodynia, hematuria, and amenorrhea [9]	Indications: blurred vision, corneal ulcer, leukoma, and poisoning [14]	A	Nervous and urinary system disorders	Five sense organ-related ailments
67	–	–	渣驯	Mineral	Mineral	Indications: hemostasis [10]	Indications: stomach and liver diseases [12]	A	Circulatory system disorders	Digestive disorders

*Shared-use medicines recorded by the 2015 edition of *China Pharmacopoeia*

Class A included the shared-use medicines with the same medicinal parts in TM and TCM; class B included the shared-use medicines with medicinal parts in TM covering that in TCM; class C included the shared-use medicines with medicinal parts in TCM covering that in TM; class D included the shared-use medicines with totally different medicinal parts in the two medical systems; and class E included the shared-use medicines with partial overlapping medicinal parts in the two medicinal systems

**Table 5:** Information on class V

No.	Family	Scientific name	Chinese name	Parts used in TCM	Parts used in TM	Uses in TCM	Uses in TM	Parts used	Disease groups treated in TCM	Disease groups treated in TM
1	Boraginaceae	*Onosma paniculatum* Bur.et Franch	滇紫草	Root	Root and root bark	Indications: macule, jaundice, hematuria, stranguria with turbid urine, constipation, and burns [20]	Indications: pneumonia, hemoptysis, measles, macule, and constipation [19]	B	Urinary system disorders	Respiratory disorders
2	Asteraceae	*Taraxacum mongolicum* Hand. -Mazz.*	蒲公英*	Whole plant	Whole plant	Indications: boils and sores, mastitis, lymphadenitis, inflammation of eyes, sore throat, lung abscess, appendicitis, jaundice caused by damp-heat, and urinary infection with difficult painful urination [10]	Indications: badkan diseases, seasonal febrile and epidemic diseases, blood disease, and *mkhris pa* diseases [15]	A	Immune system ailments, respiratory, and urinary system disorders	Immune system ailments
3	Brassicaceae	*Thlaspi arvense* L.	菥蓂	Whole plant	Seed	Indications: abdominal distension, acute appendicitis, and edema [10]	Indications: stranguria with turbid urine, liver diseases, cough, indigestion, and vomiting [14]	C	Urinary, digestive and respiratory disorders	Respiratory and digestive disorders
4	Liliaceae	*Asparagus cochinchinensis*(Lour.)Merr.	天冬	Root tuber	Root tuber	Indications: cough, thirst, sore and pain in the waist and knee, and constipation [10]	Indications: nourishing the kidney and stomach [14]	A	Respiratory and urinary system disorders	Respiratory and urinary system disorders
5	Meloidae	*Mylabris phalerata* Pallas*	斑蝥*	Whole worm	Whole worm	Indications: masses in the abdomen, cancer, chronic tinea, scrofula, vegetation, abscesses without diabrosis, malignant sore, and slough [10]	Indications: external use for carbuncles and bois, scrofula, tinea, and leukoderma; indigestion, ulcers, and abscess in the ailmentary canal when taken orally [15]	A	Circulatory system disorders	Circulatory and digestive disorders
6	Plantaginaceae	*Plantago asiatica* L.*	车前*	Seed and whole plant	Seed	Indications: edema; dysuria with difficult painful urination, diarrhea caused by summer-damp, and inflammation of the eyes; cough caused by phlegm-heat [10]	Indications: pneumonia, nephropathy, and trauma [23]	C	Urinary and respiratory disorders	Urinary and digestive disorders
7	Plantaginaceae	*Plantago depressa* Willd.*	平车前*	Seed and whole plant	Seed	Indications: edema, dysuria with difficult painful urination, diarrhea caused by summer-damp, inflammation of the eyes, and cough caused by phlegm-heat [10]	Indications: diarrhea due to cold, and dysentery [13]	C	Urinary and respiratory disorders	Urinary and digestive disorders
8	Polypodiaceae	*Drynaria roosii* Nakaike	槲蕨	Rhizome	Rhizome	Indications: rheumatic arthritis [10]	Indications: traumatic injury tinnitus, diarrhea, and alopecia [14]	A	Locomotor system ailments	Locomotor system ailments
9	Rosaceae	*Chaenomeles speciosa* (Sweet)Nakai	贴梗海棠	Fruits	Fruits	Indications: arthralgia spasm, and sore and pain in waist and knee [10]	Indications: stomach diseases, indigestion, and ulcer [14]	A	Locomotor system ailments	Digestive disorders
10	Solanaceae	*Hyoscyamus niger* L.*	天仙子*	Seed	Seed	Indications: gastric spasm and pain, asthma and cough, and mania [10]	Indications: mania, rheumatic arthritis, stomachache, chronic asthma and cough, and infectious disease [8]	A	Nervous and respiratory system ailments	Nervous and immune system ailments
11	Thymelaeaceae	*Aquilaria sinensis* (Lour.) Spreng.	白木香*	Heartwood with resin	Wood with resin	To subdue the upward qi, regulate the function of the spleen and stomach, warm the kidney, and relieve pain [10]	Indications: heart disease, adverse of qi, dyspnea, vomiting and diarrhea, hiccupping, precordial and abdominal pain with cold sensation, feeling of cold in the loins and knees in deficiency syndrome, and constipation [8]	A	Digestive disorders	Respiratory, digestive, and urinary system disorders
12	Thymelaeaceae	*Aquilariae lignum resinatum* *	沉香*	Heartwood with resin	Wood with resin	Indications: distension and pain in the chest and abdomen, vomiting or hiccupping due to cold in the stomach, and dyspnea and adverse of qi in kidney deficiency syndrome [10]	Indications: heart febrile diseases and rlung diseases [13]	A	Digestive disorders	Circulatory system disorders
13	Ursidae	*Selenarctos thibetanus* G. Cuvier	黑熊	Bile	Gall bladder, meat, and bone	Indications: infantile convulsion, epilepsy, jaundice, and external use for carbuncles, hemorrhoids, conjunctival congestion, and nebula [10]	Gall bladder: chronic ulcerated hotness, and jaundice [8] Meat: mental disease. Bone: rheumatic pain and Kaschin-Beck disease [15]	D	Nervous system ailments	Nervous system ailments
14	——	Os Draconis	龙骨	Natural mineral	Natural mineral	Indications: sweating, emission, and furuncles [9]	Indications: gall, headache, and trauma [14]	A	Nervous and immune system ailments	Nervous and immune system ailments
15	——	Borax	硼砂	Natural mineral	Natural mineral	Indications: acute tonsillitis, laryngopharyngitis, stomatitis, gingivitis, and otitis media [9]	Indications: swollen sore throat and furuncles [15]	A	Respiratory ailments	Circulatory system disorders and respiratory ailments

*Shared-use medicines recorded by the 2015 edition of *China Pharmacopoeia*

Class A included the shared-use medicines with the same medicinal parts in TM and TCM; class B included the shared-use medicines with medicinal parts in TM covering that in TCM; class C included the shared-use medicines with medicinal parts in TCM covering that in TM; class D included the shared-use medicines with totally different medicinal parts in the two medical systems; and class E included the shared-use medicines with partial overlapping medicinal parts in the two medicinal systems

Based on the plant distribution recorded in the *Flora Reipublicae Popularis Sinica* and other research on medicinal materials, the origins of the shared-use medicines could be roughly divided into the following five types: the first category included varieties that were distributed in both the Tibetan region and in the areas that were mainly covered by the Central Plains throughout history; the second category included varieties that were mainly produced in the Tibetan Plateau and its surrounding areas; the third category included varieties that were imported from South Asia, Southeast Asia, and Western Regions; the fourth category included the varieties that were generated inland and were traded in the Tibetan region; and the last category included the varieties that were mainly imported from the Han district. Detailed information was given in the Table 12.

## Results

### The similarity of medicinal parts and the efficacy of shared-use varieties used in TM and TCM

A total of 136 species of shared-use medicines that are used in prescriptions of TM and TCM were listed, and detailed information was given in Table 6 and Fig. . 1.

**Table 6:** Number of species of shared-use medicines grouped by used parts and efficacy classification

Classification	A	B	C	D	E	Total	Percentage of different efficacy groups to the total
I	17	1	0	0	1	19	14%
II	11	7	0	3	0	21	15%
III	7	1	3	3	0	14	10%
IV	48	7	6	5	1	67	49%
V	10	1	3	1	0	15	11%
Total	93	17	12	12	2	136	
Percentage of different used parts grouped in total	68%	13%	9%	9%	1%

**Fig. 1:**
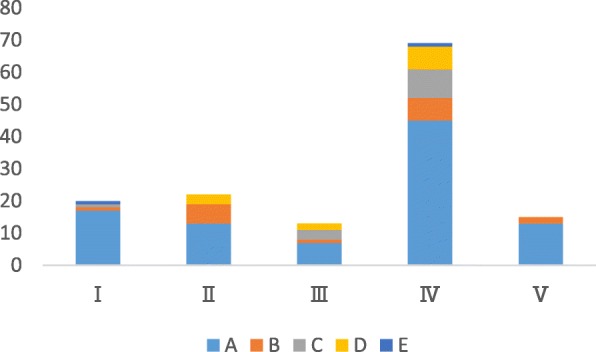
Difference in the used parts and efficacy of shared-use medicines

As shown in the above chart, nearly 50% of the shared-use medicines are used for different purposes in TM and TCM (class IV), whereas shared-use medicines with the same utilization accounted for 14% (class I), which indicated that extremely different knowledge on medicine usage existed between TM and TCM.

The proportion of shared-use medicines with similar utilizations (classes I, II, III, and V) reached 50%, which suggested a medicinal knowledge exchange between the two medical systems. For example, a number of 49 species from the inland area and 7 species from Sichuan province were recorded by the classical Tibetan medical book *Crystal Beads* [12]. However, both TM and TCM have immense diversity in their medicinal parts, and as the medical effects of one of them cover that of the other, the medicinal parts tend to be more diverse.

### The origins of shared-use varieties used in TM and TCM

According to Tables 1, 2, 3, 4 and 5, the origins of shared-use medicines used by TM and TCM were documented in Table 7.

**Table 7:** Number and percentage of different origins of shared-use medicines (classes I–V)

	I	II	III	IV	V	The total number of different origins	Percentage of different origins of shared-use medicines
The first category	6	12	8	28	13	67	49%
The second category	2	2	0	12	0	16	12%
The third category	6	4	1	8	1	20	15%
The fourth category	3	3	3	18	1	28	20%
The fifth category	2	0	2	1	0	5	4%

The first category included varieties that distributed in both Tibetan region and the areas mainly controlled by the Central Plains regime in history; the second category included varieties that were mainly produced in the Tibetan Plateau and its surrounding areas; the third category included varieties that were imported from South Asia, Southeast Asia, and Western Regions; the fourth category included the varieties that were generated in the inland and that were traded in the Tibetan region; the last category included the varieties that were mainly imported from the Han district

As shown in Table 7, the shared-use medicines that are distributed in both the Han inland and the Tibetan region accounted for the majority of the total (49%, which is the first category). Thirty-five percent of the total were imported by TM from the non-Han area through trade routes (the third category and the fourth category). Fifteen percent of the total depended on import from both the Tibetan and Han regions. Interestingly, varieties that were produced in the Tibetan region exclusively (the second category) were far more common than those that were imported from Han inland (the last category), which supported the fact that the Qinghai-Tibet Plateau also serves as the supplier for medicinal resources of TCM. The proportion of imported varieties in class I was significantly higher than that in the other groups, indicating that the shared-use medicines with the same efficacy were obviously affected by the extraterritorial medical systems. The proportion of shared-use medicines that were produced exclusively in Tibet (the second category) and the shared-use medicines that were mainly traded in the Tibetan region (the fourth category) were higher than those of other origins in class IV, which suggested a connection and an obvious evidence of independent development of the TM and TCM systems.

### The clinical application status of shared-use varieties used by TM and TCM

Since the prescription preparations are used to treat diseases in TM, the frequency of the utilization of shared-use medicines in prescription reflects its clinical application status. According to the statistical results of Tibetan medicines that are used, frequency was compiled by Zhong Guoyue [1], and the clinical application status of shared-use medicines was shown in the following Table 8.

**Table 8:** Shared-use medicines involved in TM preparations

Number of preparations involved	Scientific name of the shared-use medicines	The number of shared-use medicines/ The total number of medicinal materials[1] (percentage composition)
> 300	*Terminalia chebula* Retz. (*Terminalia chebula* Retz., *Terminalia chebula* Retz. var. *tomentella* (Kurz) C. B. Clarke), *Carthamus tinctorius* L., *Aucklandia lappa* Decne.	3/3 (100%)
201~300	PHYLLANTHI FRUCTUS, *Amomum kravanh* Pierre ex Gagnep, *Punica granatum* L., Zha-xun, *Piper longum* L, *Ewgewia caryophyllata* Thunb., MOSCHUS (*Moschus berezovskii* Flerov, *Moschus moschiferus* Linnaeus, *Mochus sifanicus* Buchner), *Inula racemosa* Hook.f., *Myristica fragrans* Houtt.	9/9 (100%)
99~200	Calcitum, BOVIS CALCULUS, *Adhatoda vasica* Nees, CINNAMOMI CORTEX, *Aquilaria agallocha* Roxb (*Aquilaria sinensis* (Lour.) Gilg, *Aquilaria agallocha* Roxb), *Zingiber officinale* Rosc., *Glycyrrhiza uralensis* Fisch. (*Glycyrrhiza uralensis* Fisch., *Glycyrrhiza inflata* Bat.), *Amomum tsaoko* Crevost et Lemair, *Styrax benzoin* Dryand., *Tinospora sinensis* (Lour.) Merr.	10/19 (53%)
51~100	BAMBUSAE CONCRETIO SILICEA (*Bambusa textilis* McClure and *Schizostachyum chinense* Rendle root exudates), *Dalbergia odorifera* T. Chen, OLIBANUM, *Rubia cordifolia* L., *Selenarctos thibetanus* G. Cuvier, *Rhodiola crenulata*(Hook. f. et Thoms.)H. Ohba , *Acorus calamus* L., *Aconitum pendulum* Busch, P*iper nigrum* L., *Crocus sativus* L., *Malva verticillata*, *Abelmoschus moschatus* Medic, *Cassia obtusifolia* L., *Tribulus terrestris* L.	14/19 (73%)
11~50	FERULAE RESINA (*Ferula fukanensis* K. M. Shen, *Ferula sinkiangensis* K. M. Shen), *Kaempferia galanga* L., *Gossampinus malabarica* (DC.) Mern, Cinnabaris, MARGARITA (*Cristaria plicata* (Leach), *Hyriopsis cumingii* (Lea)., *Pinctada martensii* (Dunker)) , Borax, Corallium	7/103 (7%)

Note: Considering that pomegranate seed rather than pomegranate is used in TM, pomegranate seeds and pomegranate were merged in this table

TCM emphasizes that the use of medicine should be compatible according to clinical needs, so the importance of shared-use medicine can be reflected by its application frequency in clinical prescriptions. Earlier, Ping [24] et al. listed 10,000 prescriptions from the First Affiliated Hospital of Guangzhou and the University of Traditional Chinese Medicine and sorted out the top 50 commonly used medicinal items, among which there are only four that were shared-use in TM and YCM, as shown in Tables 8 and 9.

**Table 9:** Shared-use medicines involved in TCM prescriptions

Medicinal materials name	Scientific name	Usage frequency	Use frequency ranking in the original text [24]
Glycyrrhizae Radix et Rhizoma	*Glycyrrhiza uralensis* Fisch., *Glycyrrhiza inflata* Bat.	65.35%	1
*Fritillaria*	*Fritillaria cirrhosa D.Don*	14.20%	11
Paeoniae radix rubra	*Paeonia veitchii Lynch*	9.76%	27
Aquilariae Lignum Resinatum	*Aucklandia lappa* Decne*.*	6.82%	48

In Tables 8 and 9, the shared-use medicines occupied a far more important place in clinical prescriptions of TM than TCM. Among the medicinal materials that were used in more than 51 Tibetan clinical prescriptions, shared-use medicines in TM and TCM accounted for 73%, and 90% of those were imported from South Asia, Southeast Asia, and Western regions. Of the top 10 medicinal materials in usage frequency, most were imported, except for musk and Zha-xun.

### Comparison of the therapeutic systems of shared-use medicines used in TM and TCM

The present study has attempted to group the shared-use medicines according to the similar types of diseases that affect the same part of the body; data was extracted from Tables 1, 2, 3, 4 and 5, as shown in Table 10.

**Table 10:** Diseases groups according to medicinal property using shared-use medicines in TCM and TM

Disease group	Number of TCM involved	Number of TM involved
Circulatory system diseases	12	13
Urinary system diseases	18	13
Immune system diseases	21	31
Nervous system diseases	23	16
Reproductive system diseases	24	21
Motion system diseases	24	14
Respiratory system diseases	36	34
Digestive system diseases	61	62

It can be easily observed from Table 10 that there was a high similarity between TM and TCM in treating digestive diseases, respiratory ailments, circulatory system diseases, and urogenital disorders. A large proportion of herbal aromatics viz., *Dalbergia odorifera* T. Chen, *Resina ferulae*, *Kaempferia galanga* L., *Piper longum* L, *Amomum kravanh Pierre ex Gagnep*, *Ewgewia caryophyllata* Thunb., *Piper nigrum L.*, *Myristica fragrans* Houtt., *Alpinia katsumadai* Hayata, *Alpinia officinarum Hance*, and *Alpinia galanga (L.) Willd* were used by both medicinal systems to treat digestive diseases, which indicated a high consensus of using volatile compounds to warm the stomach and promote digestion in TM and TCM.

### The family and genus characteristics of shared-use medicines used in TM and TCM

In the present article, we have listed a total of 136 shared-use medicines in TM and TCM, of which angiosperm, belonging to 53 families and 101 species, was used the most, accounting for 71% of the total 136 types of shared-use medicines. Leguminosae was the most widely used family, followed by Zingiberaceae, Umbelliferae, Compositae, Liliaceae, Polygonaceae, Labiatae, Thymelaeaceae, and Ranunculaceae. Three pteridophytes (having two families and three species) were used in both TM and TCM. Sixteen animal resources and 12 mineral resource medicines were used, accounting for 12% and 9%, respectively, of the total. The high proportion is due to the wide use of animal and mineral resource medicines in TM. Detailed information was given in Table 11.

**Table 11:** Number of species used in shared-use medicines

Classification	Family	No. of species used
Angiosperm	Leguminosae	9
	Zingiberaceae	8
	Apiaceae	7
	Asteraceae	6
	Liliaceae	4
	Polygonaceae	4
	Labiatae	3
	Thymelaeaceae	3
	Ranunculaceae	3
	others	54
Gymnosperm	Polypodiaceae	2
	Selaginellaceae	1
Resinae	–	5
Animalia	–	16
Mineral group	–	12
Others	–	4

## Discussion

It is interesting and complicated to discuss the relationships between the two neighboring traditional systems of medicine and their long histories. Naive materialism is the foundation of both; meanwhile, TM is largely influenced by Tibetan Buddhist culture while TCM is largely influenced by Confucian culture. In terms of medical theory and knowledge on medicinal materials, the two-, three-, five-, and six-group methods are widely used in both TM and TCM, such as the “five essences” (water, fire, soil, qi, air) of the former and the “five elements” (gold, wood, water, soil, fire) of the latter. There is a high similarity in knowledge of the two medicinal systems on properties and flavors of medicine; for example, both styles of medicine are grouped according to the properties of “cold and warm,” and “sour, bitter, sweet, pungent, and salty” are used both in the “six flavors theory” of TM and the “five flavors theory” of TCM. However, the present paper discussed the relationship of TM and TCM in terms of the similarity and dissimilarity of efficacy and the origins of shared-use medicines, instead of the philosophical theory.

The possible reasons for the similarity of different medical systems in the usage of medicine could be the following:

1. Significant pharmacological effects of medicine;
2. Mutual communication between the two medical systems about clinical practices;
3. Same influence of other traditional systems of medicine;
4. Coincidence.

While the reason for different medicinal properties of the same medicine varies, it could be due to the following:

1. Different medicinal parts or preparation used;
2. Different medicinal prescriptions;
3. Distinctive local culture and heritage of knowledge on medicinal application;
4. Diverse regional common ailments as well as natural and socioeconomic conditions;

Based on the research results, this article has drawn the following inferences.

### Medicinal knowledge exchange occurred during the parallel development of TM and TCM

## Shared-use medicines are mostly determined by flora similarity and medicinal trade

It can be observed in the Table 12 that a total of 67 shared-use medicines were distributed in both the Tibetan and Han regions, accounting for 49% of the total. The Huaxia people originated in northwestern China, rising north of the Yangtze River and belonging to the China-Japan forest subregion. While the Tibetans rose in the Qinghai-Tibetan Plateau, where the eastern part belongs to the China-Himalayan ecological subregion, and the west is the Qinghai-Tibetan Plateau plant subregion. There is a large crossover in plant varieties between the two ethnic regions [25]. In comparing the shared-use medicines listed in Table 9 with common plants in Northern China [26] and the Tibetan regions [27], Compositae, Leguminosae, Gramineae, Ranunculaceae, Labiatae, Umbelliferae, Liliaceae, and Rosaceae are common families within both regions. Therefore, the large number of cross-plant species formed by flora is the main reason for the large number of shared-use medicines in the two traditional systems of medicine.

**Table 12:** Historical origins of shared-use medicines (classes I–V)

Origins	I (A total of 19)	II (A total of 21)	III (A total of 14)	IV (A total of 68)	V (A total of 15)
The first category	*Bungarus multicinctus* Blyth, *Cuscuta chinensids* Lam., *Rheum palmatum* L., *Selaginella pulvinata* (Hook. et Grev.) Maxim., Z*ingiber officinale* Rosc., Honeybee	*Allium sativum* L., *Coriandrum sativum* L., *Euphorbia fischeriana* Steudel, *Aconitum pendulum* Busch, *Cervus nippon* Temminck, *Cervus elaphus* Linnaeus, *Rubia cordifolia* L., *Trigonella ruthenica* L., *Trogopterus xanthipes* Milne-Edwards, *nula helenium* L., *Malva verticillata*, *Raphanus sativus* L.	*Polygonatum sibiricum* Delar. ex Redouté, *Stellera chamaejasme* Linn., Sulfur, *Rana tamporaria chensinensis* David, *Juglans regia* L., *Rumex nepalensis* Spreng., *Urtica laetevirens* Maxim., *Lycium chinense* Mill.	*Acorus calamus* L, Actinolite Asbestos., *Bergenia purpurascens* (Hook. f. et Thoms.) Engl, BOVIS CALCULUS, Calamina, Calcitum, Cinnabaris, Gypsum Fibrosum, Haematitum, *Notopterygium forbesii* de Boiss., MAGNETITUM, *Fraxinus stylosa* Lingelsh., Natrii Sulfas, *Notopterygium forbesii* de Boiss., *Phytolacca acinosa* Roxb., PYRITUM, *Pyrrosia lingua* (Thunb.) Farw., REALGAR, Succinite (Amber), *Tribulus terrestris* L., Zha-xun, *Paeonia veitchii* Lynch, *Lagenaria siceraria* (Molina) Standl., *Lagopsis supine* (Steph. ex Willd.)Ikonn.-Gal. ex Knorr, *Leonurus japonicus* Thunb. (Leonurus japonicas Houtt.), *Leonurus sibiricus* L., *Equus asinus* Linnaeus, *Dendrobium hookerianum* Lindl.	*Asparagus cochinchinensis* (Lour.)Merr., *Chaenomeles speciosa* (Sweet)Nakai, *Drynaria fortunei*(Kunze) J.Sm., *Hyoscyamus niger* L., *Taraxacum mongolicum* Hand. -Mazz., Borax, *Onosma paniculatum* Bur.et Franch, *Plantago asiatica* L., *Plantago depressa* Willd., T*hlaspi arvense* L., Os Draconis, *Selenarctos thibetanus* G. Cuvier, M*ylabris phalerata* Pallas.
The second category	*Ophiocordyceps sinensis*, *Rheum tanguticum* Maxim. ex Regel.	RHODIOLAE CRENULATAE RADIXET RHIZOMA, *Vladimiria souliei* (Franch.)Ling.	–	*Arenaria kansuensis* Maxim, *Fritillaria cirrhosa* D.Don, N*ardostachys chinensis* Batal., *Nardostachys jatamansi* (D. Don) DC., *Notopterygium incisum* Ting ex H. T. Chang, S*aussurea laniceps* Hand.-Mazz., *Gentiana crassicaulis* Duthie ex B u rk, *Gentiana straminea* Maxim, *Moschus berezovskii* Flerov, *Mochus sifanicus* Buchner, *Moschus moschiferus* Linnaeus, *Sinopodophyllum emodii* (wall) Ying.	–
The third category	*Aucklandia lappa* Decne., *Dalbergia odorifera* T. Chen, F*erula sinkiangensis* K. M. Shen, *Ferula fukanensis K*. M. Shen, *Kaempferia galanga* L., *Piper longum* L.	*Amomum kravanh* Pierre ex Gagnep, *Ewgewia caryophyllata* Thunb., *Piper nigrum* L., *Myristica fragrans* Houtt.	*Areca catechu* L	*Boszvellia carterii* Birdw, *Crocus sativus* L., *Dryobalanops aromatica* Gaertn. f., *Strychnos nuxvomica* L., *Styrax benzoin* Dryand., *Terminalia chebula* Retz., *Terminalia chebula* Retz. var. *tomentella* (Kurz) C. B. Clarke, *Santalum album* L.	*Aquilaria agallocha* Roxb
The fourth category	*Alpinia katsumadai* Hayata, *Alpinia officinarum* Hance, *Amomum tsaoko* Crevost et Lemair.	*Alpinia galanga* (L.) Willd, *Sesamum indicum* L., *Aristolochia debilis* Sieb. et Zucc.	*Glycyrrhiza uralensis* Fisch., *Glycyrrhiza inflata* Bat., *Trigonella foenum-grecum* L.	*Acacia catechu* (L.f.)Willd, *Adhatoda vasica* Nees, BAMBUSAE CONCRETIO SILICEA*, *Carthamus tinctorius* L., *Cassia obtusifolia* L., *Cristaria plicata* (Leach), *Hyriopsis cumingii* (Lea)., *Pinctada martensii*(Dunker), *Curcuma longa* L., *Gossampinus malabarica* (DC.)Mern, *Pterocarpus indicus* Willd., *Saccharum officinarum* L, *Abrus precatorius* L, *Vitis vinifera* L., *Punica granatum* L., *Sapindus mukorossi* Gaertn., T*inospora sinensis* (Lour.) Merr., *Abelmoschus moschatus* Medic.	*Aquilaria sinensis* (Lour.) Gilg

The first category included varieties that were distributed in both the Tibetan region and the areas that were mainly controlled by the Central Plains regime in history; the second category included varieties that were mainly produced in the Tibetan Plateau and its surrounding areas; the third category included varieties that were imported from South Asia, Southeast Asia and Western Regions; the fourth category included the varieties that were generated in the inland and which were traded in the Tibetan region; the last category included the varieties that were mainly imported from the Han district.

As shown in the Table 12, 56 taxa of shared-use medicines were imported from the non-Han region to the Tibetan region, accounting for 35% of the total. On the other hand, 20 taxa were imported from the non-Tibetan region to Han inland, accounting for 15% of the total. This suggests that imported medicinal materials from the southern regions have some influence on the two medicinal systems, especially on TM (from Table 7), and that both TM and TCM have closely communicated with other extraterritorial medical systems and acquired practical experience regarding the usage of medicine.

1. *The shared-use medicines marked significant differences in their medicinal properties*

In the tang dynasty, the central plains and Tubo exchanged information closely, according to the edited books and research articles. It is generally believed that TM has been largely influenced by TCM, which is reflected in the pulse diagnosis and visceral knowledge of TM [28]. However, this paper showed that shared-use medicines used in TM and TCM marked significant differences in their medicinal properties, and most of the widely used Tibetan medicines were imported from the non-Han area. The unique medicinal use of TM is reflected in earlier books, such as *Yutu Materia Medica*, *Tara Materia Medica*, and *Miaoyin Materia Medica*. Based on the analysis, this article believed that medicinal materials and medicinal use experiences of TM were mainly summarized by its clinical practitioners during and before the Tubo dynasty in the extreme natural conditions in the Tibetan plateau. TM developed in parallel with TCM and was greatly influenced, especially in medicinal resources by traditional medicinal systems in southern Asia in the later stage.

As for the shared-use medicines distributed in the Tibetan plateau listed in the Table 12, although they are local products, varieties such as *Notopterygium incisum* Ting ex H. T. Chang, Gentianae Macrophyllae Radix, Moschus, and Gansong, were recorded in the ancient traditional Chinese medicine and were traded to the Han region through the western Sichuan Plateau, which was controlled for a long time by the Central Plains dynasticism since the Qin and Han dynasties [29], as well as the southern Gansu province. For example, the use of Snow Lotus Herb [30] can be traced back to Qing dynasty in *Supplements to Compendim of Materia Medica*, which appears to be a teaching from Uygur Medicine, while the use of *Arenaria kansuensis* Maxim has been recorded in *The Collection of Chinese Herbal Medicines* 1975 version, which appears to be a teaching from folk practices. Therefore, the Tibetan plateau serves as a medicinal resource for TCM.

In summary, shared-use medicines by TM and TCM marked a significant difference in medicinal properties.

2. *Shared-use medicines with similar medicinal properties presented an obvious commercial characteristic of materia medica as well as the same chemical profile*

It can be observed in Table 7 that, out of 19 shared-use medicines with similar medicinal effects (class I), a number of 13 were traded medicines, up to 68% of the total, which was much higher than other proportions. These varieties have marked an obvious commodity attribute. For example, RHEI RADIX ET RHIZOMA was commonly traded through the silk road. *Ophiocordyceps sinensis* was the representative example of traded medicine from the Tibetan region to Han inland, which was originally recorded in the ancient Tibetan medicinal book *Ten Million Buddhist Relics* by Suka·Nii6anmuduoji (1439–1475). It was not traded in the Han region until the Kangxi period and was recorded in *A General Description of SiChuan* in the Yongzheng period. *Rhodiola crenulata* (Hook. f. et Thoms.) H. Ohba was recorded in *Chinese Pharmacopoeia* in the year of 1977 as commonly used Tibetan medicine, while currently it is widely used by traditional Chinese medicine.

As shown in the Table 12, among the shared-use medicines that are used in TM and TCM, there was a large number of aromatic medicines that are rich in volatile compounds. Those aromatic medicines are used to warm the stomach and promote digestion in both TM and TCM, which is closely related to the pharmacological activity of volatile components.

### Tibetan plateau not only provides medicinal usage knowledge of TCM, but it also serves as a supply of medicinal resources attributing to “high altitude” locations

Tibetan culture exchanged medicinal information closely with Han culture officially during the Tang dynasty. After the perdition of the Tubo regime, the culture exchange moved towards the flock through the tea-horse ancient road. Medicines as *Ophiocordyceps sinensis*, Rhei Radix Et Rhizoma, *Nardostachys chinensis* Batal., Gentianae Macrophyllae Radix, *Fritillaria cirrhosa* D. Don, *Notopterygium incisum* Ting ex H. T. Chang, and Moschus were imported from the Tibetan plateau and were widely used in TCM. It can be observed in the Table 12 that a number of 16 traditional Chinese medicines were imported from the Tibetan plateau, which was much higher than the Tibetan medicines that were imported from the Han region (five species). The traditional Chinese medicines that were imported from the Tibetan plateau could be divided into the following three categories: the first category, such as *Notopterygium incisum* Ting ex H. T. Chang, *Fritillaria*, Moschus, and Gentianae Macrophyllae Radix, after a long history of medicinal use in TCM, were used quite differently in TM; the second category, such as *Ophiocordyceps sinensis* and Rhodiolae Crenulatae Radixet Rhizoma, were used similarly in TM and TCM, because they were imported during the near ancient time or even in modern times; the last category, such as the Snow Lotus Herb, presented a different medicinal effect from TM, because of the influence of other traditional medicinal systems. In summary, the Tibetan plateau not only provides medicinal usage knowledge of TCM, but it also serves as a supply of medicinal resources. According to the literature, the western Sichuan and western Yunnan regions were the main trade routes. In contrast, few Tibetan medicines were imported from the Han region, and no available ancient literature could provide a clue that TM has learned knowledge from TCM. Therefore, this paper believed that TM started to acquire medicinal knowledge from TCM only in modern times. For example, *Fritillaria cirrhosa* D. Don has been used in TM to stop coughs, as it is used in TCM, only since modern times.

Attributed to its unique geographical location and cultural diversity, the Tibetan region plays a role as a development cradle for various traditional medical theories and knowledge

Tibetan culture has been extensively and deeply influenced by many ancient civilizations of the world. It has been exchanging knowledge with ancient Indian, Central Plains and Persian cultures for a long time. During the Hellenistic period after Alexander’s expedition, it was inevitably influenced by the Mediterranean through ancient India. There are not many existing cultures like Tibetan culture that are influenced by multi-mainstream cultures from the ancient world. It also shows a stark contrast to the demised history of various cultures in the neighboring Western Regions.

Due to the religious influences and relatively closed environment, the social development of areas in the Tibetan region has been slow since the “Peihong period” of Tibetan Buddhism, thus providing conditions for the preservation and inheritance of medicinal knowledge. Taking the *Terminalia chebula* Retz., which is used most commonly in TM, as an example, its prescription “Da San Guo Tang” came from India and is still commonly used in a Tibetan compound recipe. In contrast, after the Ming dynasty, *Terminalia chebula* Retz. var. *tomentella* (Kurz) C. B. Clarke and Phyllanthi Fructus were almost discarded from use in TCM prescriptions. Tibetan medicine Zota and some other varieties are similar examples of this case. Therefore, we believe the medicinal distinctness of the Tibetan plateau has been preserved since the “Xiang Xiong” period, and foreign medicines, including *Terminalia chebula* Retz., *Aucklandia lappa* Decne., and *Carthamus tinctorius* L., have also been promoted in TM. This can be demonstrated by the special status of exotic species in Tibetan medicinal prescriptions.

Research results of the present study are scientific and representative

To verify the scientific research results, this study also compared 3107 TM varieties and more than 2200 kinds of TCM varieties according to the *Dictionary of Chinese National Medicine* and *The Collection of Chinese Herbal Medicines*. A total of 313 shared-use medicines were documented, and the proportions of each variety are 12% for class I, 12% for class II, 12% for class III, 60% for class IV, and 50% for class V, which was consistent with the results of this paper, thus indicating the representativeness of the statistical results of this study (Table 13).

**Table 13:** Shared-use medicines that are involved in more than 51 TM preparations

Efficacy classification	Shared-use medicines
Class I	*Aucklandia lappa* Decne., PHYLLANTHI FRUCTUS, *Alpinia katsumadai* Hayata, *Piper longum* L, *Amomum tsaoko* Crevost et Lemair, *Dalbergia odorifera* T. Chen, *Rhodiola crenulata* (Hook. f. et Thoms.) H. Ohba, *Piper nigrum* L.
Class II	*Myristica fragrans* Houtt., ZINGIBERIS RHIZOMA, *Ewgewia caryophyllata* Thunb., *Rubia cordifolia* L., *Malva verticillata.*
Class III	*Glycyrrhiza uralensis* Fisch., *Glycyrrhiza inflata* Bat.*.*
Class IV	*Terminalia chebula* Retz., *Carthamus tinctorius* L., MOSCHUS *(Moschus berezovskii* Flerov, *Moschus moschiferus* Linnaeus, *Mochus sifanicus* Buchner*)*, Calcitum, BOVIS CALCULUS, *Adhatoda vasica* Nees, CINNAMOMI CORTEX, *Punica granatum* L., *Styrax benzoin* Dryand., *Tinospora sinensis* (Lour.) Merr., BAMBUSAE CONCRETIO SILICEA (*Bambusa textilis* McClure and *Schizostachyum chinense* Rendle root exudates), OLIBANUM, *Selenarctos thibetanus* G. Cuvier, *Aconitum pendulum* Busch, *Crocus sativus* L., *Abelmoschus moschatus* Medic, *Cassia obtusifolia* L., *Tribulus terrestris* L.
Class V	*Aquilaria agallocha* Roxb

## Conclusions

The present study was based on the statistical analysis of the authoritative publications and papers of national medicine and traditional Chinese medicine. To reflect the exact clinical use status of TM and TCM and to make sure the results are representative, clinical formulas of TM were used for statistics.

Studies showed that medicinal knowledge exchange occurred during the parallel development of TM and TCM, the shared-use medicines are mostly determined by the flora similarity and medicinal trade and marked significant differences in their medicinal properties, and shared-use medicine with similar medicinal properties presented an obvious commercial characteristic of materia medica, as well as the same chemical profile. From the breed point of view, Tibetan plateau not only provides medicinal usage knowledge of TCM, but it also serves as a supply of medicinal resources, attributing to the “high altitude” influence. Attributed to its unique geographical location and cultural diversity, the Tibetan region plays a role as a development cradle for various traditional medical theories and knowledge.

## Data Availability

Not applicable.

## Abbreviations

TCM: Traditional Chinese medicine
TM: Tibetan medicine
